# First-line drugs for hypertension

**DOI:** 10.1590/S1516-31802010000100012

**Published:** 2010-01-07

## Abstract

**BACKGROUND:**

Sustained elevated blood pressure, unresponsive to lifestyle measures, leads to a critically important clinical question: What class of drug to use first-line? This review answers that question.

**OBJECTIVES:**

Primary objective: To quantify the benefits and harms of the major first-line anti-hypertensive drug classes: thiazides, beta-blockers, calcium channel blockers, angiotensin converting enzyme (ACE) inhibitors, alpha-blockers, and angiotensin II receptor blockers (ARB).

**SEARCH STRATEGY:**

Electronic search of MEDLINE (Jan. 1966-June 2008), EMBASE, CINAHL, the Cochrane clinical trial register, using standard search strategy of the hypertension review group with additional terms.

**SELECTION CRITERIA:**

Randomized trials of at least one year duration comparing one of 6 major drug classes with a placebo or no treatment. More than 70% of people must have blood pressure ≥ 140/90 mmHg at baseline.

**DATA COLLECTION AND ANALYSIS:**

The outcomes assessed were mortality, stroke, coronary heart disease (CHD), cardiovascular events (CVS), decrease in systolic and diastolic blood pressure, and withdrawals due to adverse drug effects. Risk ratio (RR) and a fixed effects model were used to combine outcomes across trials.

**MAIN RESULTS:**

Of 57 trials identified, 24 trials with 28 arms, including 58,040 patients met the inclusion criteria. Thiazides (19 RCTs) reduced mortality (RR 0.89, 95% CI 0.83, 0.96), stroke (RR 0.63, 95% CI 0.57, 0.71), CHD (RR 0.84, 95% CI 0.75, 0.95) and CVS (RR 0.70, 95% CI 0.66, 0.76). Low-dose thiazides (8 RCTs) reduced CHD (RR 0.72, 95% CI 0.61, 0.84), but high-dose thiazides (11 RCTs) did not (RR 1.01, 95% CI 0.85, 1.20). Beta-blockers (5 RCTs) reduced stroke (RR 0.83, 95% CI 0.72, 0.97) and CVS (RR 0.89, 95% CI 0.81, 0.98) but not CHD (RR 0.90, 95% CI 0.78, 1.03) or mortality (RR 0.96, 95% CI 0.86, 1.07). ACE inhibitors (3 RCTs) reduced mortality (RR 0.83, 95% CI 0.72-0.95), stroke (RR 0.65, 95% CI 0.52-0.82), CHD (RR 0.81, 95% CI 0.70-0.94) and CVS (RR 0.76, 95% CI 0.67-0.85). Calcium-channel blocker (1 RCT) reduced stroke (RR 0.58, 95% CI 0.41, 0.84) and CVS (RR 0.71, 95% CI 0.57, 0.87) but not CHD (RR 0.77 95% CI 0.55, 1.09) or mortality (RR 0.86 95% CI 0.68, 1.09). No RCTs were found for ARBs or alpha-blockers.

**AUTHORS’ CONCLUSIONS:**

First-line low-dose thiazides reduce all morbidity and mortality outcomes. First-line ACE inhibitors and calcium channel blockers may be similarly effective but the evidence is less robust. First-line high-dose thiazides and first-line beta-blockers are inferior to first-line low-dose thiazides.

Wright JM, Musini VM. First-line drugs for hypertension. Cochrane Database of Systematic Reviews 2009, Issue 3. Art. No.: CD001841. DOI: 10.1002/14651858.CD001841.pub2.

FURTHER INFORMATION: Centro Cochrane do Brasil Rua Pedro de Toledo, 598 Vila Clementino — São Paulo (SP) — Brasil CEP 04039-001 Tel. (+55 11) 5579-0469/5575-2970 http://www.centrocohranedobrasil.org.br/

This section was edited under the responsibility of Brazilian Cochrane Centre

The full review is available (free access) from http://cochrane.bvsalud.org/portal/php/index.php?lang=pt.

